# A role for mutations in *AK9* and other genes affecting ependymal cells in idiopathic normal pressure hydrocephalus

**DOI:** 10.1073/pnas.2300681120

**Published:** 2023-12-15

**Authors:** Hong Wei Yang, Semin Lee, Bethany C. Berry, Dejun Yang, Shaokuan Zheng, Rona S. Carroll, Peter J. Park, Mark D. Johnson

**Affiliations:** ^a^Department of Neurological Surgery, University of Massachusetts Chan Medical School, Worcester, MA 01655; ^b^Brigham and Women’s Hospital, Boston, MA 02115; ^c^Harvard Medical School, Boston, MA 02115; ^d^Department of Neurological Surgery, University of Massachusetts Memorial Health, Worcester, MA 01655

**Keywords:** AK9, whole exome sequencing, motile cilia, normal pressure hydrocephalus

## Abstract

Idiopathic normal pressure hydrocephalus (iNPH) is an enigmatic neurological disorder that develops after age 60 and is characterized by gait difficulty, dementia, and incontinence. Recently, we reported that heterozygous *CWH43* deletions may cause iNPH. Here, we identify mutations affecting nine additional genes (*AK9*, *RXFP2, PRKD1, HAVCR1, OTOG, MYO7A, NOTCH1, SPG11, and MYH13*) that are statistically enriched among iNPH patients. The encoded proteins are all highly expressed in choroid plexus and ependymal cells, and most have been associated with cilia. Damaging mutations in *AK9*, which encodes an adenylate kinase, were detected in 9.6% of iNPH patients. Mice homozygous for an iNPH-associated *AK9* mutation displayed normal cilia structure and number, but decreased cilia motility and beat frequency, communicating hydrocephalus, and balance impairment. *AK9*+/− mice displayed normal brain development and behavior until early adulthood, but subsequently developed communicating hydrocephalus. Together, our findings suggest that heterozygous mutations that impair ventricular epithelial function may contribute to iNPH.

## Significance

Idiopathic normal pressure hydrocephalus (iNPH) is characterized by ventriculomegaly, imbalance, incontinence, and dementia, but the causes are poorly understood. We previously reported that *CWH43* deletions may cause iNPH. Here, we identify nine additional genes harboring mutations that are statistically enriched among iNPH patients. Most of these genes have been associated with cilia and are highly expressed in the ciliated ventricular epithelium. Damaging mutations in *AK9*, which encodes an adenylate kinase, were detected in 9.6% of iNPH patients. Mice harboring *AK9* mutations displayed decreased cilia beat frequency, communicating hydrocephalus, and balance impairment. *AK9*+/− mice displayed normal brain development and behavior until early adulthood, but subsequently developed hydrocephalus. Thus, mutations that impair ventricular epithelial function may play a causal role in iNPH.

Idiopathic normal pressure hydrocephalus (iNPH) is a form of adult-onset communicating hydrocephalus that is characterized by enlarged cerebral ventricles, gait difficulty, incontinence, and cognitive impairment (1). It is a common neurological disorder that usually develops after the age of 60 (2). It has been estimated that 1.4 to 2.9% of the population over the age of 65, 5.9% of the population over the age of 80, and nearly 1 in 7 nursing home residents have iNPH (3–7).

The etiology and pathophysiology of iNPH are poorly understood. iNPH has been associated with hypertension, hypercholesterolemia, diabetes, and alcohol use (2, 8–10), but the physiological mechanisms underlying these associations are unclear. Ventricular CSF stasis, abnormal cerebrovascular reactivity, and increased amplitude of intracranial pressure waves have all been observed in iNPH patients, but the underlying mechanisms are not known (11–13).

Several reports of familial iNPH have been published in the literature (including pedigrees with autosomal dominant transmission), suggesting a possible genetic origin (14–18). A study of one family with a history of familial iNPH identified a heterozygous mutation in *CFAP43* (Cilia And Flagella Associated Protein 43) in two family members as a possible cause of familial iNPH (14). The Cfap43 protein is important for the normal structure and function of motile cilia and sperm flagella, and homozygous deletion of this gene in mice causes abnormalities in motile cilia structure, infertility, and hydrocephalus in an autosomal recessive manner. A recent single nucleotide polymorphism study reported intronic copy number loss in the *SFMBT1* gene in 26% of shunt-responsive iNPH patients compared to 4.2% of the general population, but the significance of this finding was not determined (19). We recently identified recurrent heterozygous deletions in *CWH43* in 15% of patients with sporadic iNPH (20). *CWH43* encodes a protein that modifies the lipid anchor of GPI-anchored proteins. Mice that are heterozygous for one of the iNPH-associated *CWH43* mutations develop an iNPH-like syndrome that includes enlarged ventricles, abnormalities of gait and balance, and a decrease in the number of ventricular cilia.

Here, we report the identification of heterozygous iNPH-associated mutations in nine additional genes that collectively account for about 50% of shunt-responsive iNPH cases. All of the encoded proteins are highly expressed in multiciliated choroid plexus and ependymal cells, and most have previously been associated with cilia function. Genetically engineered mice that are heterozygous for an iNPH-associated damaging mutation in *AK9* (which encodes adenylate kinase 9) appear normal and are fertile at birth, but develop communicating hydrocephalus and imbalance as they age. Brain structure and ependymal motile cilia structure are normal, but ependymal motile cilia function is compromised due to decreased motility. Taken together with recent reports from our laboratory and others indicating roles for heterozygous mutations in *CWH43* (20) and *CFAP43* (14) in normal pressure hydrocephalus, our findings implicate heterozygous damaging mutations in a number of different genes that impair multiciliated ventricular cell function as potential contributors to adult-onset iNPH.

## Methods

### Patients.

All experiments involving human subjects were approved by the UMass Chan Institutional Review Board and by the Institutional Review Board at Brigham and Women’s Hospital. Fifty-three unrelated patients who presented to a neurosurgical clinic with unexplained complaints of ventricular enlargement, gait difficulty, incontinence, and/or cognitive decline underwent an evaluation that included a history, neurological examination, and cranial imaging. Patients were consented for the study prior to CSF drainage. A trial of lumbar CSF drainage was then performed, and those who improved were selected for shunt placement. Only those patients who also improved after ventriculoperitoneal shunt placement were included for whole exome analysis. Quantitative measurements of gait speed, stride length, and performance on the Timed Up and Go (TUG) test, as well as patient and caregiver reports of changes in urinary incontinence, were used to assess symptoms before and after CSF drainage as we have described previously (20).

### Whole Exome Sequencing and Data Analysis.

The 53 patients with shunt-responsive iNPH were enrolled and analyzed in three separate cohorts (n = 20, n = 12, and n = 21). Genomic DNA was isolated from whole blood and submitted in three independent batches for whole exome sequencing (50X coverage, 150 bp paired-end sequencing, Illumina HiSeq 2000). Single nucleotide variants (SNVs) and insertions/deletions (indels) were identified (Human Genome build GRCh37, bwa-mem, Genome Analysis Toolkit HaplotypeCaller). Genetic alterations with a frequency greater than 1% in a public database containing over 60,000 genomes [ExAC database (21)] were excluded. The minor allele frequency (MAF) of each mutation in the study group was compared to that in the general population (i.e., the combined MAF across all ethnic groups, ExAC database), and statistical enrichment among iNPH patients was calculated using the two-tailed *X*^2^ test with and without the Yates correction. Four publicly available computer prediction algorithms (SIFT, PROVEAN, MutationTaster, and PolyPhen-2) were used to predict the effect of each mutation on protein function. Genes with three or more mutations that were predicted to be damaging by at least two of the four computer prediction algorithms were selected for further study. Only genes harboring damaging mutations in at least two of the three patient cohorts were included for the final analysis. Genetic alterations were confirmed using PCR and Sanger sequencing. The whole exome sequencing data from normal pressure hydrocephalus patients are available in dbGaP https://www.ncbi.nlm.nih.gov/projects/gapprev/gap/cgi-bin/study.cgi?study_id=phs002296.v1.p1 (22). The accession ID is phs002296.v1.p1.

### In Situ mRNA Hybridization.

In situ mRNA hybridization images for iNPH-associated genes were obtained from a public database [Allen Brain Atlas (23)].

### Immunohistochemistry.

Mouse brains were fixed in 4% paraformaldehyde for 24 h, infiltrated by immersion in a solution containing 15% sucrose followed by 30% sucrose, and snap frozen in Tissue Plus O.C.T (Fisher Scientific). Sagittal or coronal cryostat sections of mouse brains were prepared and stained for fluorescence immunohistochemistry using antibodies directed against Cwh43 (1:250, HPA048140, Sigma-Aldrich), Ak9(1:250, HPA030804, Sigma-Aldrich), HAVCR1(1:500, 14791, Cell signaling), Rxfp2 (1:500, sc-374293, Santa Cruz Bioecology), otogelin (1:200, ARP65594, Aviva Systems Biology), notch 1 receptor (1:500, MA5-11961, ThermoFisher Scientific), protein kinase D1(1:200, sc-638, Santa Cruz Bioecology), myosin heavy chain 13 (1:500, PA5-89942, ThermoFisher Scientific), myosin VIIA (Myo7a) (1:200, HPA028918-, Sigma-Aldrich), spatacsin (SAB3500003, Sigma-Aldrich), or acetylated α-tubulin (1:1,000, sc-23950, Santa Cruz Bioecology). Nuclei were counterstained using DAPI.

### Primary Ependymal Cell Culture.

Ependymal cells were harvested by microdissection from the brains of newborn wild-type mice, and the cells were trypsinized for 10 min at 37 °C with 0.01% trypsin, followed by centrifugation for 5 min at 900 g. The cells were then suspended mechanically in DMEM containing 100 U/mL penicillin, 100 μg/mL streptomycin, and 10% fetal calf serum (FCS). Dissociated cells from a single brain were seeded in two laminin-coated (10 μg/mL) wells of a 24-multiwell plate and maintained in DMEM-10% FCS in a humidified 5% CO_2_ atmosphere at 37 °C for 2 d, and the medium was then changed to DMEM with 2% FCS for an additional 10 to 14 d, at which time motile cilia could readily be observed under the microscope. The cells were then fixed in paraformaldehyde, stained using antibodies directed against Ak9, Rxfp2, Cwh43, myo7a, or acetylated α-tubulin (to visualize cilia), and imaged using fluorescence confocal microscopy.

### RT-PCR.

Total RNA was extracted from the brain, ependymal layer, liver, kidney, trachea, testis, and sperm of WT mice, and one-step RT-PCR for the two major AK9 isoforms was performed. The primers used were forward 5′-ggaggatggattgtggagaa-3′; reverse 5′-ggatctagtgccctctgctg-3′ for isoform 1 (NCBI Reference Sequence: XM_030245201.1) and forward 5′-ccctgatcaaggcaatgaat-3′; reverse 5′-ttctgggatttgggtctctg-3′ for isoform 2 (NCBI Reference Sequence: XM_006512939.3). The PCR products were separated on a 1.2% agarose gel to visualize alternatively spliced transcripts of *AK9*.

### Generation and Analysis of AK9 Mutant Mice.

All experiments and procedures involving mice were approved by the University of Massachusetts Chan Medical School Institutional Animal Care and Use Committee (IACUC). All mice were housed on a 12:12 light/dark cycle. Mice were housed in autoclaved static mouse cages with filtered lids on ¼ inch corn cob bedding with nesting materials. Sterilized bottles were filled with triple-filtered acidified water. The room was set at 30 to 70% humidity and 68 to 79 °F. C57bl6 mice harboring an iNPH-associated *AK9* mutation (coinciding to the human iNPH-associated *AK9* deletion T4119A:p.Y1373X) were generated using a CRISPR/Cas9 approach in collaboration with the Mutagenesis Core and Transgenic Core Facility at the UMass Chan Medical School. Mice were bred to generate heterozygous (*AK9^WT/1373X^*) and homozygous (*AK9^1373X/1373X^*)animals. The donor sequence for the mutation is T​TCA​ATC​GTA​CTG​CAG​CTA​AGC​GAA​GGA​GAG​ACA​ATC​AAA​CCT​GTT​GAA​AAT​GCA​GAG​AAt​CCG​CTG​tAa​CCT​GTA​ATC​CAT​CGT​CAC​TAC​ATC​TAT​TTCTTATCTAATAAGCAAACTAAAGAGAAATTT. This mutation generates a stop codon and leads to a truncated Ak9 protein lacking the C-terminal kinase domain. The genotyping was done by PCR using primers 5′ GCAGCTAAGCGAAGGAGAGA-3′ and 5′-TCATAATCCGCACAGGCATA-3′, followed by direct sequencing. Both male and female mice were used for experiments.

In some cases, the brains of WT, *AK9^WT/1373X^,* or *AK9^1373X/1373X^* were harvested, and the lateral ventricular surface was fixed for examination using immunohistochemistry or electron microscopy.

### MRI.

3D spin echo (SE) T2-weighted MR images of the brains of *AK9^WT/WT^*, *AK9^WT/1373X^* or *AK9^1373X/1373X^* mice were acquired on a Philips Ingenia CX dStream 3.0 T system at The Advanced MRI Center, UMass Chan Medical School. A custom-made solenoid T/R coil with the following imaging parameters is used: 32 slices with slice thickness of 0.25 mm; field of view (FOV) of 20 mm × 18 mm with matrix size of 80 × 72 (reconstruction matrix = 160 × 160); TR/TE = 2,000/80 ms; flip angle (FA) = 90°; TSE-factor = 8; NSA = 2. Ventricular volume was calculated using ImageJ and a custom automated computer algorithm implemented with MatLab code (MathWorks).

### Rotarod Test.

Strength, balance, and coordination in mice were assessed by the rotarod performance test using a commercially available rotarod testing system (Harvard Apparatus). Mice (7 *Ak9^WT/WT^* males, *7 AK9^WT/1373X^* males, *4 AK9^1373X/1373X^* females, and 1 *AK9^1373X/1373X^* male) were placed on a rotating dowel that was programmed to accelerate from 4 to 40 rpm over 5 min. The elapsed time before falling off the dowel was recorded automatically. The maximum trial time was 5 min. All animals underwent one training session followed by three experimental trials per session. Mice were allowed to rest for at least 15 min between trials. The mean balance time for the three trials was calculated and used for further analyses. Animal weight was recorded at the conclusion of each testing session. Statistical significance for laboratory studies was calculated using the two-tailed unpaired *t* test with a significance threshold of *P* < 0.05.

### Analysis of Cilia Beat Frequency and Sperm Movement.

Mice were euthanized using CO_2_ according to the AVMA Guidelines, the brains were harvested, and 1-mm-thick brain slices containing the lateral ventricular wall were harvested acutely by sectioning the brain using an Acrylic Adult Mouse Brain Slicer Matrix through the bregma. Brain slices from wild-type and *AK9* mutant mice were maintained in vitro in phosphate-buffered saline (pH 7.4) at room temperature. Ependymal motile cilia beat frequency was assessed by highspeed video microscopy with light interferometry using ImageJ. Mouse sperm from wild-type and *AK9* mutant mice were harvested, and flagellar motility was assessed in vitro using high-speed video microscopy and ImageJ software.

### Functional Analysis of Ak9 Kinase Activity.

Site-specific mutagenesis was used to generate a series of expression vectors encoding human *AK9* fused to an amino-terminus His-tag and harboring each of the iNPH-associated *AK9* mutations. An in vitro TnT® Coupled Reticulocyte Lysate System (Promega) was then used to express mRNA from the 1 mg of Ak9 wild-type or mutant plasmid and translate that mRNA into Ak9 wild-type and mutant proteins according to the manufacturer’s protocol. One quarter of the expressed Ak9 protein lysate was used for western blot confirmation, and the other three quarters were passed through a HisPur™ Ni-NTA Spin Column (ThermoScientific). Adenosine diphosphate (ADP) was then added to the lysate, and adenylate kinase activity was measured via luminometric detection of adenosine triphosphate (ATP) production (Abcam, ab228557, USA). In some cases, the expressed Ak9 proteins were immunopurified using an anti-His antibody, and the purified protein was used to perform in vitro kinase assays measuring the conversion of ADP to ATP.

## Results

### Patient Characteristics.

Whole exome sequencing of DNA obtained from 53 patients with shunt-responsive iNPH was performed in three independent cohorts as we have described previously (20). Collectively, there were 29 females and 24 males. The median age at the time of presentation was 75 y (range 65 to 89 y). All of the patients had gait difficulty and enlarged cerebral ventricles out of proportion to the degree of brain atrophy. Urinary incontinence and cognitive impairment were present in 79% and 83% of the patients, respectively. All of the patients experienced an improvement in their symptoms after lumbar CSF drainage and again after ventriculoperitoneal shunt placement, indicating the presence of shunt-responsive idiopathic normal pressure hydrocephalus.

### Identification of iNPH-Associated Genes.

We analyzed sequencing data as described in the *Methods* and selected genes that met the following criteria: 1) at least three mutations that were statistically enriched when compared to the general population (*X*^2^ with Yates correction, *P* < 0.05), and 2) mutations predicted to be damaging by at least two of four computer prediction algorithms. We performed a Permutation Test using 1,000 permuted control genomes and our 53 genomes to determine how much each gene was selected relative to random mutations. After excluding common single nucleotide polymorphisms (SNPs), the average number of single nucleotide variations (SNVs) in each sample was 11,199 and was roughly equivalent among patients and controls. Permutation *P* values for the iNPH-associated mutations ranged from *P* < 0.001 to *P* < 0.012 for all cases. A total of 10 genes (including *CWH43*) met these criteria. All of the mutations were heterozygous, and 44% were recurrent (Table 1). All 10 genes displayed damaging mutations in at least two of three independent cohorts of 12 to 21 patients each, and 6 of the 10 genes displayed damaging mutations in all three cohorts (*SI Appendix*, Fig. S1). This was observed despite the fact that none of the mutations occurs at a frequency greater than 1 in 175 in the general population. Our finding of heterozygous mutations is consistent with either an autosomal dominant mode of transmission [as has been reported for familial iNPH (14, 18)] or digenic transmission.

**Table 1.** Genes showing enrichment for recurrent damaging mutations in patients with shunt-responsive iNPH

Variant type	Gene mutation	Exp allele freq	Obs allele freq	Chi square (Yates Corr)	PROVEAN	SIFT	MutationTaster	PolyPhen-2
**Nonsyn SNV**	**PRKD1:NM_002742:exon1:c.T20C:p.L7P**	**0.003**	**0.0377**	***P*** **< 0.0001**	**B**	**D**	**D**	**B**
**Nonsyn SNV**	**PRKD1:NM_002742:exon1:c.T20C:p.L7P**	**0.003**	**0.0377**	***P*** **< 0.0001**	**B**	**D**	**D**	**B**
**Nonsyn SNV**	**PRKD1:NM_002742:exon1:c.T20C:p.L7P**	**0.003**	**0.0377**	***P*** **< 0.0001**	**B**	**D**	**D**	**B**
**Nonsyn SNV**	**PRKD1:NM_002742:exon1:c.T20C:p.L7P**	**0.003**	**0.0377**	***P*** **< 0.0001**	**B**	**D**	**D**	**B**
**Nonsyn SNV**	**RXFP2:NM_001166058: exon8:c.A664C:p.T222P**	**0.00488**	**0.0377**	***P*** **< 0.0001**	**D**	**B**	**D**	**D**
**Nonsyn SNV**	**RXFP2:NM_001166058: exon8:c.A664C:p.T222P**	**0.00488**	**0.0377**	***P*** **< 0.0001**	**D**	**B**	**D**	**D**
**Nonsyn SNV**	**RXFP2:NM_001166058: exon8:c.A664C:p.T222P**	**0.00488**	**0.0377**	***P*** **< 0.0001**	**D**	**B**	**D**	**D**
**Nonsyn SNV**	**RXFP2:NM_001166058: exon8:c.A664C:p.T222P**	**0.00488**	**0.0377**	***P*** **< 0.0001**	**D**	**B**	**D**	**D**
**FS Del**	**CWH43:NM_001286791: exon16:c.2005delA:p.K669fs**	**0.00569**	**0.0377**	***P*** **< 0.0002**	**NA**	**NA**	**D**	**NA**
**FS Del**	**CWH43:NM_001286791: exon16:c.2005delA:p.K669fs**	**0.00569**	**0.0377**	***P*** **< 0.0002**	**NA**	**NA**	**D**	**NA**
**FS Del**	**CWH43:NM_001286791: exon16:c.2005delA:p.K669fs**	**0.00569**	**0.0377**	***P*** **< 0.0002**	**NA**	**NA**	**D**	**NA**
**FS Del**	**CWH43:NM_001286791: exon16:c.2005delA:p.K669fs**	**0.00569**	**0.0377**	***P*** **< 0.0002**	**NA**	**NA**	**D**	**NA**
**Nonsyn SNV**	**OTOG:NM_001277269: exon28:c.C3466T:p.R1156W**	**0**	**0.0009**	***P*** **< 0.0001**	**D**	**B**	**D**	**B**
**Nonsyn SNV**	**OTOG:NM_001277269: exon33:c.G4240A:p.D1414N**	**0**	**0.0188**	***P*** **< 0.0001**	**B**	**D**	**D**	**D**
**Nonsyn SNV**	**OTOG:NM_001277269: exon 33:c.G4240A:p.D1414N**	**0**	**0.0188**	***P* < 0.0001**	**B**	**D**	**D**	**D**
**Nonsyn SNV**	**OTOG:NM_001277269:** **exon44:c.C7570T:p.R2524C**	**0.01757**	**0.018** **8**	***P*** **< 0.0035**	**D**	**D**	**P**	**D**
**Nonsyn SNV**	**OTOG:NM_001277269: exon44:c.C7570T:p.R2524C**	**0.01757**	**0.0188**	***P* < 0.0035**	**D**	**D**	**P**	**D**
**Nonsyn SNV**	**HAVCR1:NM_012206:exon3:c.C652T:p.P218S**	**0.0000281**	**0.0094**	***P*** **< 0.0001**	**B**	**D**	**D**	**D**
**Nonsyn SNV**	**HAVCR1:NM_012206:exon2:c.G317A:p.R106H**	**0.00017**	**0.0094**	***P*** **< 0.0001**	**D**	**D**	**D**	**D**
**FS Ins**	**HAVCR1:NM_012206: exon2:c.148_149insAG:p.G50fs**	**0.00224**	**0.0183**	***P*** **< 0.0001**	**NA**	**NA**	**D**	**NA**
**FS Ins**	**HAVCR1:NM_012206: exon2:c.148_149insAG:p.G50fs**	**0.00224**	**0.0183**	***P*** **< 0.0001**	**NA**	**NA**	**D**	**NA**
**Nonsyn SNV**	**MYH13:NM_003802:exon30:c.C4142T:p.T1381M**	**0.000099**	**0.0094**	***P*** **< 0.0001**	**D**	**D**	**D**	**D**
**Nonsyn SNV**	**MYH13:NM_003802:exon19:c.G2174A:p.R725Q**	**0**	**0.0094**	***P*** **< 0.0001**	**D**	**D**	**D**	**D**
**Nonsyn SNV**	**MYH13:NM_003802:exon19:c.C2119T:p.R707W**	**0.0002**	**0.0094**	***P*** **< 0.0035**	**D**	**D**	**D**	**D**
**Nonsyn SNV**	**MYH13:NM_003802:exon3:c.C174G:p.D58E**	**0**	**0.0094**	***P*** **< 0.0001**	**B**	**D**	**D**	**B**
**Nonsyn SNV**	**MYO7A:NM_001127179: exon25:c.A3166G:p.K1056E**	**0**	**0.0094**	***P*** **< 0.0001**	**D**	**B**	**D**	**D**
**Nonsyn SNV**	**MYO7A::NM_001127180: exon29:c.C3659T:p.P1220L**	**0.000096**	**0.0094**	***P*** **< 0.0001**	**D**	**D**	**D**	**D**
**stopgain**	**MYO7A::NM_001127180: exon32:c.G4293A:p.W1431X**	**0**	**0.0094**	***P*** **< 0.0001**	**NA**	**NA**	**D**	**NA**
**Nonsyn SNV**	**MYO7A::NM_001127180: exon38:c.C5113T:p.R1705W**	**0**	**0.0094**	***P*** **< 0.0001**	**D**	**D**	**P**	**D**
**stopgain**	**AK9:NM_001145128:exon32:c.T4119A:p.Y1373X**	**0**	**0.0094**	***P*** **< 0.0001**	**NA**	**NA**	**D**	**NA**
**Nonsyn SNV**	**AK9:NM_001145128:exon28:c.G3476C:p.R1159P**	**0**	**0.0094**	***P*** **< 0.0001**	**D**	**D**	**D**	**ND**
**Nonsyn SNV**	**AK9:NM_001145128:exon31:c.C4022T:p.T1341I**	**0**	**0.0094**	***P*** **< 0.0001**	**D**	**B**	**D**	**ND**
**Nonsyn SNV**	**AK9::NM_001145128:exon12:c.C1138T:p.R380C**	**0.000026**	**0.0094**	***P*** **< 0.0001**	**D**	**D**	**D**	**D**
Nonsyn SNV	AK9:NM_001145128:exon36:c.T4961C:p.F1654S	0.000067	0.0094	*P* < 0.0001	B	D	P	B
**Nonsyn SNV**	**SPG11:NM_025137:exon15:c.A2833G:p.R945G**	**0**	**0.0094**	***P*** **< 0.0001**	**D**	**D**	**D**	**D**
**Nonsyn SNV**	**SPG11:NM_025137:exon5:c.G946C:p.D316H**	**0**	**0.0094**	***P*** **< 0.0001**	**D**	**D**	**D**	**D**
**Nonsyn SNV**	**SPG11:NM_025137:exon30:c.G5489A:p.G1830D**	**0.000033**	**0.0094**	***P*** **< 0.0001**	**D**	**D**	**D**	**D**
Nonsyn SNV	SPG11:NM_025137:exon1:c.G44A:p.G15D	0.0001082	0.0094	*P* < 0.0035	B	B	P	B
**Nonsyn SNV**	**NOTCH1:NM_017617: exon28:c.G5224T:p.A1742S**	**0.0005135**	**0.0094**	***P*** **< 0.1051**	**B**	**D**	**D**	**B**
**Nonsyn SNV**	**NOTCH1:NM_017617: exon26:c.G4988A:p.R1663Q**	**0.0000369**	**0.0094**	***P*** **< 0.0001**	**B**	**B**	**D**	**D**
**Nonsyn SNV**	**NOTCH1:NM_017617: exon25:c.G4222A:p.E1408K**	**0**	**0.0094**	***P*****<** **0.0001**	**D**	**D**	**D**	**D**
Nonsyn SNV	NOTCH1:NM_017617: exon18:c.C2877G:p.N959K	0.0001048	0.0094	*P* < 0.0001	B	B	D	B

Mutations in bold lettering are predicted to be damaging. *=*P* < 0.05 without Yates correction. *P* > 0.05 due to low number of alleles analyzed in the ExAC database. Nonsyn SNV, nonsynonymous single nucleotide variant; FS Del, frameshift deletion; FS Ins, frameshift insertion; B, benign; D, damaging; P, polymorphism; ND, not determined.

Next, we correlated the iNPH-associated genetic alterations with clinical and demographic variables. Mutations in one or more of these 10 genes were present in 58% of the patients, and there was limited overlap among them (*SI Appendix*, Fig. S1). Nine of 53 iNPH patients (17%) harbored mutations affecting two or more iNPH-associated genes (*SI Appendix*, Fig. S1). We observed strong trends toward increased cognitive dysfunction and increased hypertension among patients for whom no recurrent mutations have yet been identified (*P* = 0.057 and *P* = 0.057, respectively, two-tailed Proportion Test). Given the reported association between hypertension and iNPH (9, 10), this finding raises the possibility that iNPH patients with hypertension or severe cognitive impairment may represent a clinically distinct subgroup in which iNPH arises via a hypertension-related mechanism.

Structural analysis of the encoded proteins indicated that the mutations tended to occur in or near functional domains. This was consistent with computer algorithm predictions that the iNPH-associated mutations are function-altering mutations (*SI Appendix*, Fig. S2).

### Brain Expression of iNPH-Associated Genes.

**Fig. 1.**
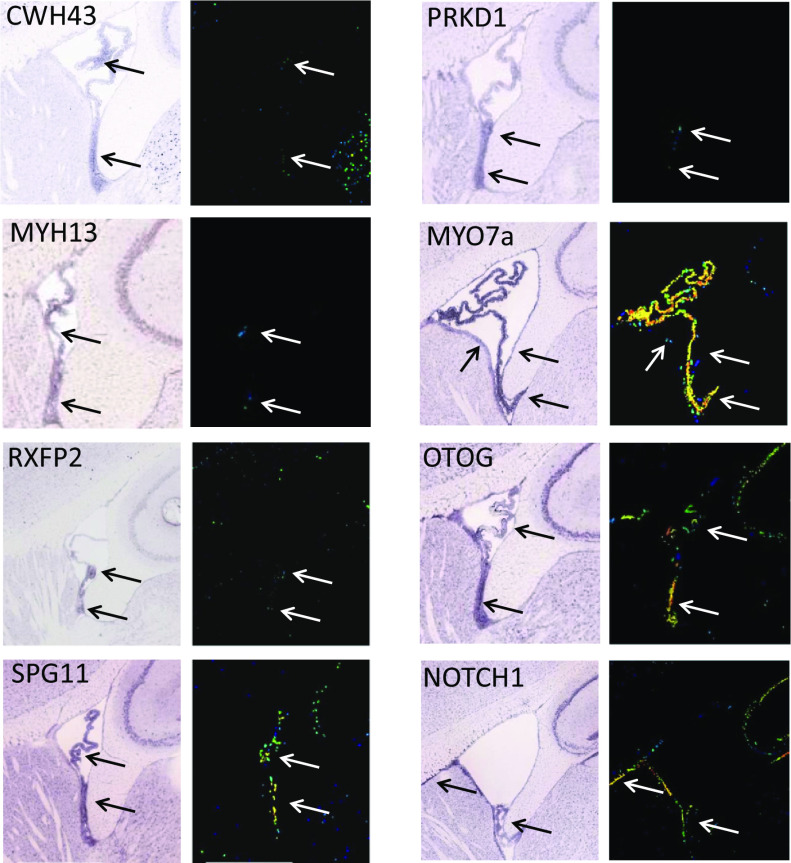
iNPH-associated mRNA expression in the ventricular region of the mouse brain. In situ hybridization showing expression of mRNAs for eight iNPH-associated genes in the choroid plexus and ventricular ependyma of the mouse brain. Both light and pseudocolor expression intensity images are shown. Arrows identify hybridization signal in the mouse choroid plexus and ependyma. Data are obtained from the Allen Brain Atlas.

A literature review indicated that 7 of the 10 iNPH-associated genes that we identified have previously been associated with cilia function (*PRKD1, OTOG, NOTCH1, MYO7A,* and *HAVCR1*) (24–29) or hydrocephalus (*AK9* and *SPG11*). Analysis of mRNA in situ hybridization images obtained from a public database [Allen Brain Atlas (23)] revealed increased mRNA expression for 8 of 10 iNPH-associated genes in the mouse ventricular ependyma or choroid plexus (Fig. 1). In situ mRNA hybridization data for the remaining genes, *AK9* and *HAVCR1*, were unavailable.

**Fig. 2.**
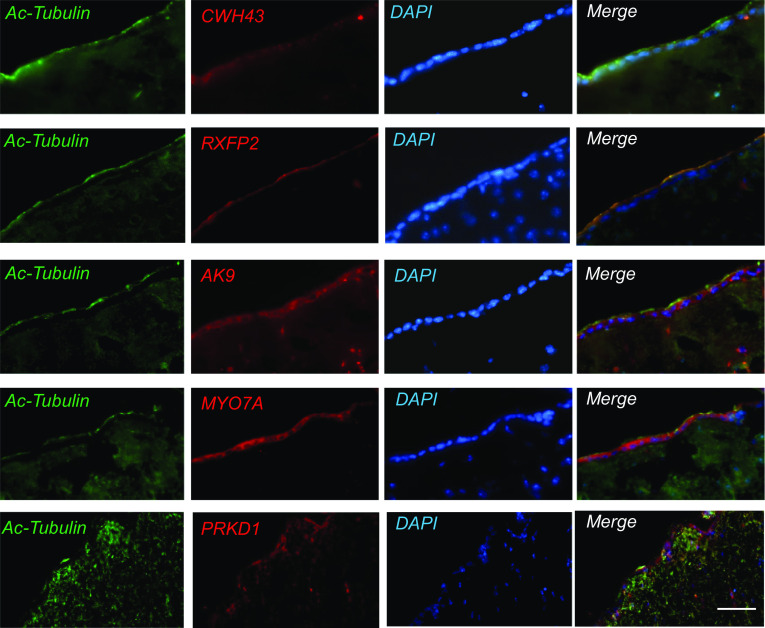
Expression of iNPH-associated proteins in the ventricular region of the mouse brain. Immunofluorescence micrographs showing immunoreactivity for proteins expressed from iNPH-associated genes in the ependymal layer and subventricular zone of mice. Immunohistochemistry for Cwh43, PRKD1, Rxfp2, Ak9, and Myo7a was performed using cryostat sections from 7-wk-old C57BL6 mice. Cilia were visualized using an antibody directed against acetylated α-tubulin (Ac-tubulin). Nuclei were stained with DAPI. (Scale is 25 µm.)

**Fig. 3.**
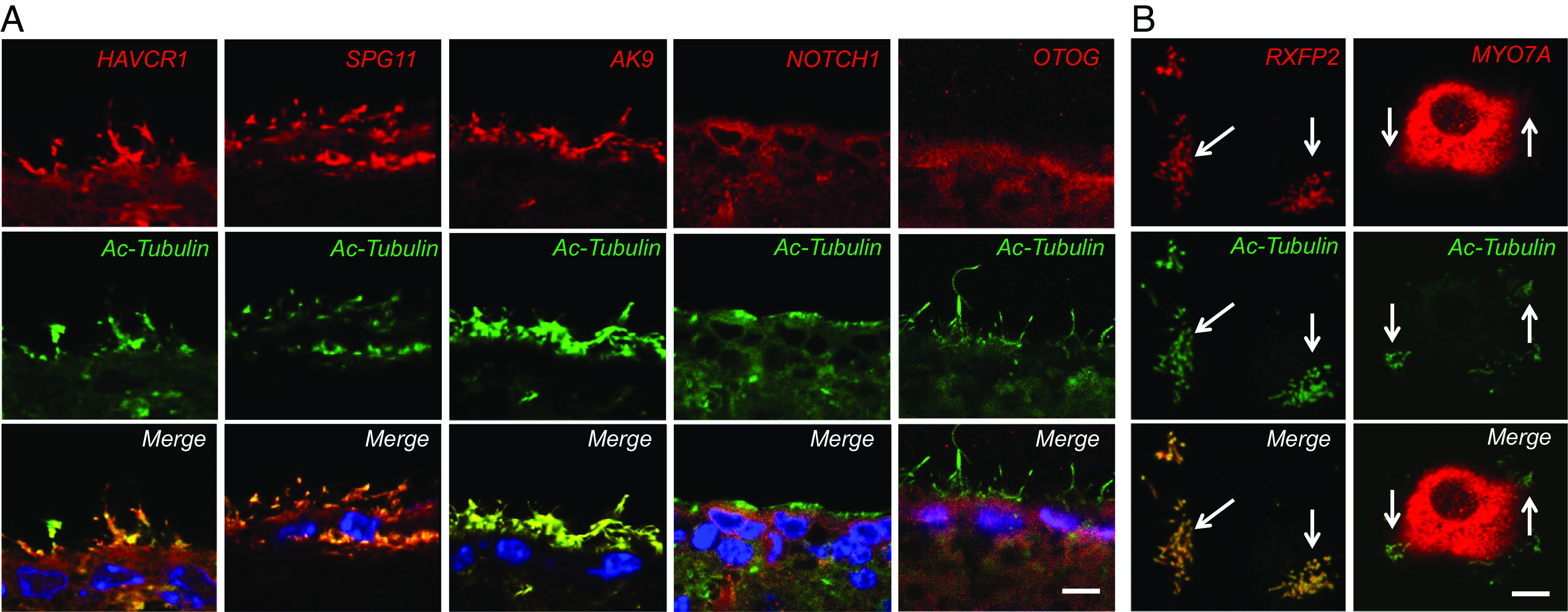
Relationship between proteins from iNPH-associated genes and ependymal cilia. (*A*) High resolution immunohistochemistry showing localization of SPG11/spatacsin, Ak9, HAVCR1/Kim1, Notch1, and otogelin immunoreactivity in ependymal cells. Cilia were visualized using an acetylated α-tubulin antibody (green). (Scale is 5 µm.) (*B*) Immunocytochemistry for Rxfp2 or Myo7a (red) in cultured mouse ependymal cells. Cilia were visualized using an acetylated α-tubulin antibody (green). Arrows show areas where iNPH-associated protein immunoreactivity colocalizes with cilia. (Scale is 5 µm.)

Using frozen sections of the adult mouse brain, we performed immunocytochemistry for proteins encoded by *PRKD1, MYO7A, AK9, CWH43, OTOG, NOTCH1, HAVCR1, SPG11, MYH13,* and *RXFP2* and found that all showed increased expression in the ciliated choroid plexus and ventricular ependyma (Figs. 2 and 3). We also examined whether the iNPH-associated proteins are localized to cilia in vivo or in cultured mouse ependymal cells using fluorescence immunocytochemistry and confocal microscopy (Fig. 3). Six of the iNPH-associated proteins examined (Cwh43, Rxfp2, Ak9, HAVCR1/Kim1, spatacsin, and myo7A) localized to ependymal motile cilia in vivo or in cultured mouse ependymal cells (Fig. 3).

### iNPH-Associated Mutations in AK9 Are Damaging Mutations.

We previously reported the identification of recurrent heterozygous *CWH43* deletions in 15% of patients with shunt-responsive iNPH and showed that genetically engineered mice that were heterozygous or homozygous for one of these iNPH-associated *CWH43* deletions develop an iNPH-like syndrome characterized by communicating hydrocephalus and impairments in gait and balance (20). In the current study, we identified heterozygous mutations in *AK9* in 5 of the 53 iNPH patients (9.6%). *AK9* encodes adenylate kinase 9, which is a nucleoside mono- and diphosphate kinase involved in nucleoside homeostasis (30). Using ATP as a phosphate donor, Ak9 catalyzes the phosphorylation of AMP, dAMP, CMP, and dCMP to generate the corresponding nucleoside diphosphates. When GTP is the phosphate donor, only AMP and CMP are phosphorylated. In addition to the generation of nucleoside diphosphate products, a nucleoside diphosphate kinase (NDPK) activity was also present with subsequent formation of nucleoside triphosphates (ATP, CTP, GTP, UTP, dATP, dCTP, dGTP, and TTP). Nucleoside diphosphates and triphosphates are essential for ciliary or flagellar motility.

RT-PCR of mRNA obtained from mouse testis, sperm, kidney, and trachea revealed two major *AK9* transcripts that were differentially expressed in a tissue-specific manner. Ependymal cells uniquely expressed isoform 2 as the predominant isoform (*SI Appendix*, Fig. S3). Immunohistochemistry revealed high levels of *AK9* expression in multiciliated ependymal cells and choroid plexus cells of the mouse brain, as well as in the head and flagellum of sperm. A review of public databases [the Human Protein Atlas (31)] revealed high *AK9* protein expression in several other tissues, including the testis, pituitary gland, respiratory epithelium, and fallopian tube.

**Fig. 4.**
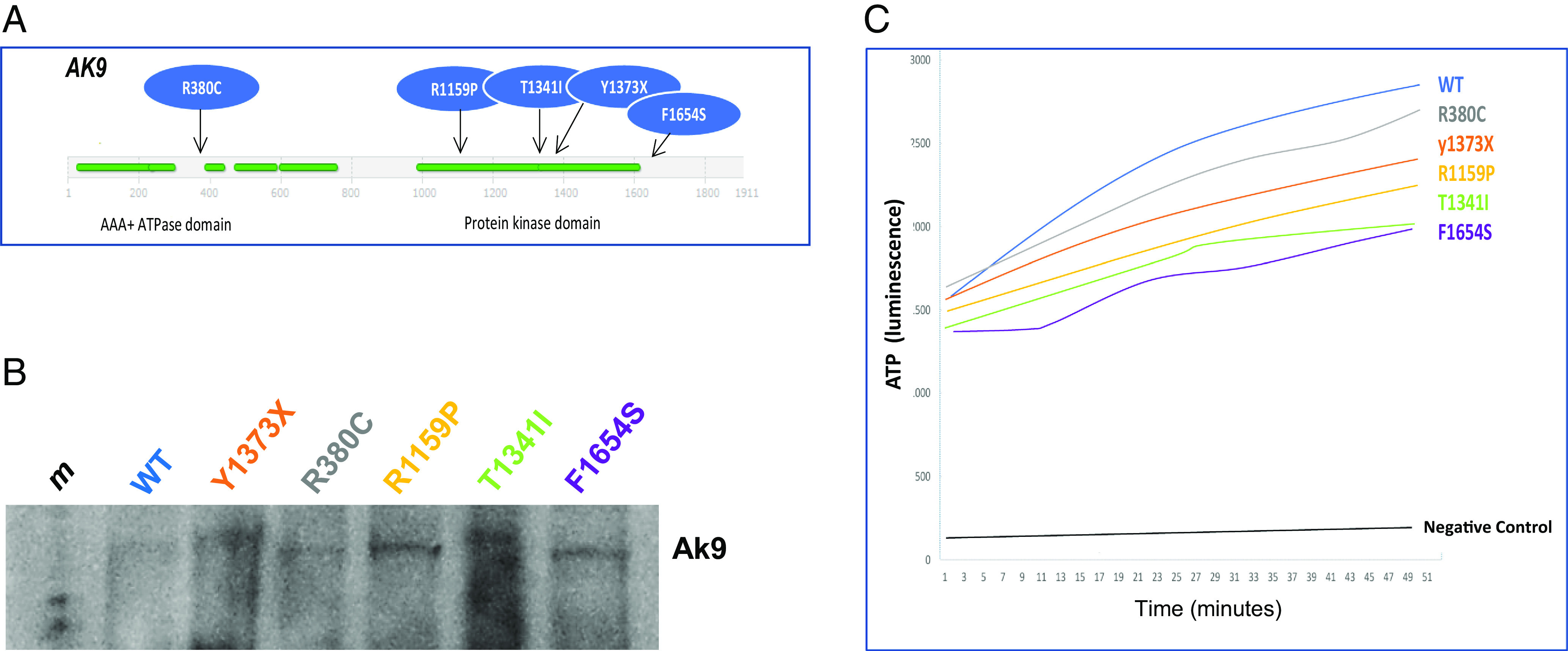
iNPH-associated *AK9* mutations are damaging mutations. (*A*) Schematic diagram showing domain structure of Ak9 and location of iNPH-associated *AK9* mutations. (*B*) Western blot showing expression of WT Ak9 protein and Ak9 protein harboring iNPH-associated mutations. Expression vectors encoding GFP (control), wild-type human Ak9 (WT), or human Ak9 proteins harboring one of the iNPH-associated *AK9* mutations were used to express the encoded control or mutant proteins in a cell-free in vitro transcription and translation system. (*C*) In vitro kinase assays were performed to measure the conversion of ADP to ATP by recombinant WT Ak9 and Ak9 harboring iNPH-associated mutations. ATP was detected using a spectrophotometric method. Recombinant Ak9 proteins were expressed using equal amounts of cDNA in a cell-free in vitro transcription and translation system.

Multiple computer algorithms predicted that four of the five *AK9* mutations were damaging. These mutations were clustered in the region of the third kinase domain of the Ak9 protein (Fig. 4*A*). To determine the effect of these mutations on Ak9 activity, we first used site-specific mutagenesis to generate a series of expression vectors encoding human Ak9 harboring an amino-terminus His-tag and each of the iNPH-associated *AK9* mutations. We then used an in vitro translation system to express each of the encoded Ak9 wild-type or mutant proteins (Fig. 4*B*) and measured their ability to convert ADP to ATP in in vitro kinase assays. As shown in Fig. 4*C*, all of the iNPH-associated *AK9* mutations decreased Ak9 activity when compared to the wild-type protein, confirming that they were indeed loss of function mutations.

### iNPH-Associated AK9 Mutation Decreases Sperm Flagellar Motility.

Using a CRISPR/Cas9 approach, we generated mice harboring one of the iNPH-associated mutations (Y373X) identified in this study. This mutation creates a stop codon that leads to a truncated form of Ak9 which lacks the third ATPase domain and the C-terminal end of the protein. Male and female C57bl6 *AK9*+/− heterozygous mice appeared grossly normal, fertile, and displayed a normal life span. Female *AK9*−/− homozygous mice also appeared grossly normal and had a normal lifespan, but produced litters with a smaller number of pups than wild-type mice.

**Fig. 5.**
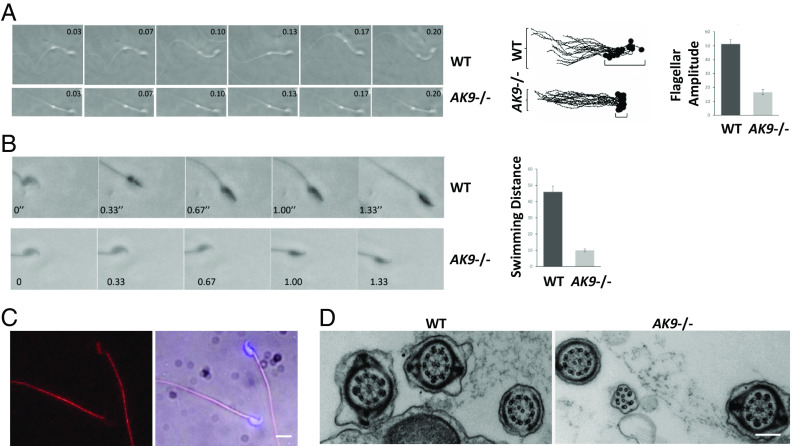
*AK9* mutation decreases sperm motility and progression. (*A*) (*Left*) Time lapse video microscopy images demonstrating flagellar excursion of mouse sperm obtained from wild-type (WT) and *AK9*−/− mice. (*Middle*) Superimposed camera lucida drawings of WT and *AK9*−/− mouse sperm illustrating flagellar excursion and sperm head forward progression. (*Right*) Quantification of flagellar motion data shown in *Left* and *Middle* panels. (*P* < 0.01, unpaired *t* test). (*B*) (*Left*) Time lapse video microscopy images demonstrating motility and progression of mouse sperm obtained from WT and *AK9*−/− mice. (*Right*) Quantification of sperm progression data shown in the *Left* panel. (*P* < 0.001, unpaired *t* test). (*C*) Fluorescence and light micrographs showing Ak9 immunoreactivity (red) in WT mouse sperm. (Scale is 5 µm.) (*D*) Transmission electron micrographs showing the structure of flagella from WT and *AK9*−/− mouse sperm. (Scale is 0.5 µm.)

Male *AK9*−/− homozygous mice were infertile. Video microscopy of sperm obtained from male *AK9*−/− mice revealed normal sperm number, but markedly decreased flagellar excursion (Fig. 5*A*) and severely impaired swimming ability (Fig. 5*B*) when compared to sperm from wild-type or *AK9*+/− mice. Immunocytochemistry revealed strong Ak9 immunoreactivity in both the tail and the head of wild-type mouse sperm (Fig. 5*C*). To determine whether iNPH-associated *AK9* mutation affected flagellar structure, we examined the sperm of wild-type mice and *AK9*−/− mice using transmission electron microscopy. The diameter, 9 + 2 structure and dynein arms of the flagella were normal in *AK9*−/− mouse sperm (Fig. 5*D*), suggesting that the decreased interconversion of nucleoside phosphates caused by the iNPH-associated *AK9* mutation affected flagellar motility but not structure.

### Heterozygous AK9 Mutations Cause Adult-Onset Communicating Hydrocephalus in Mice.

**Fig. 6.**
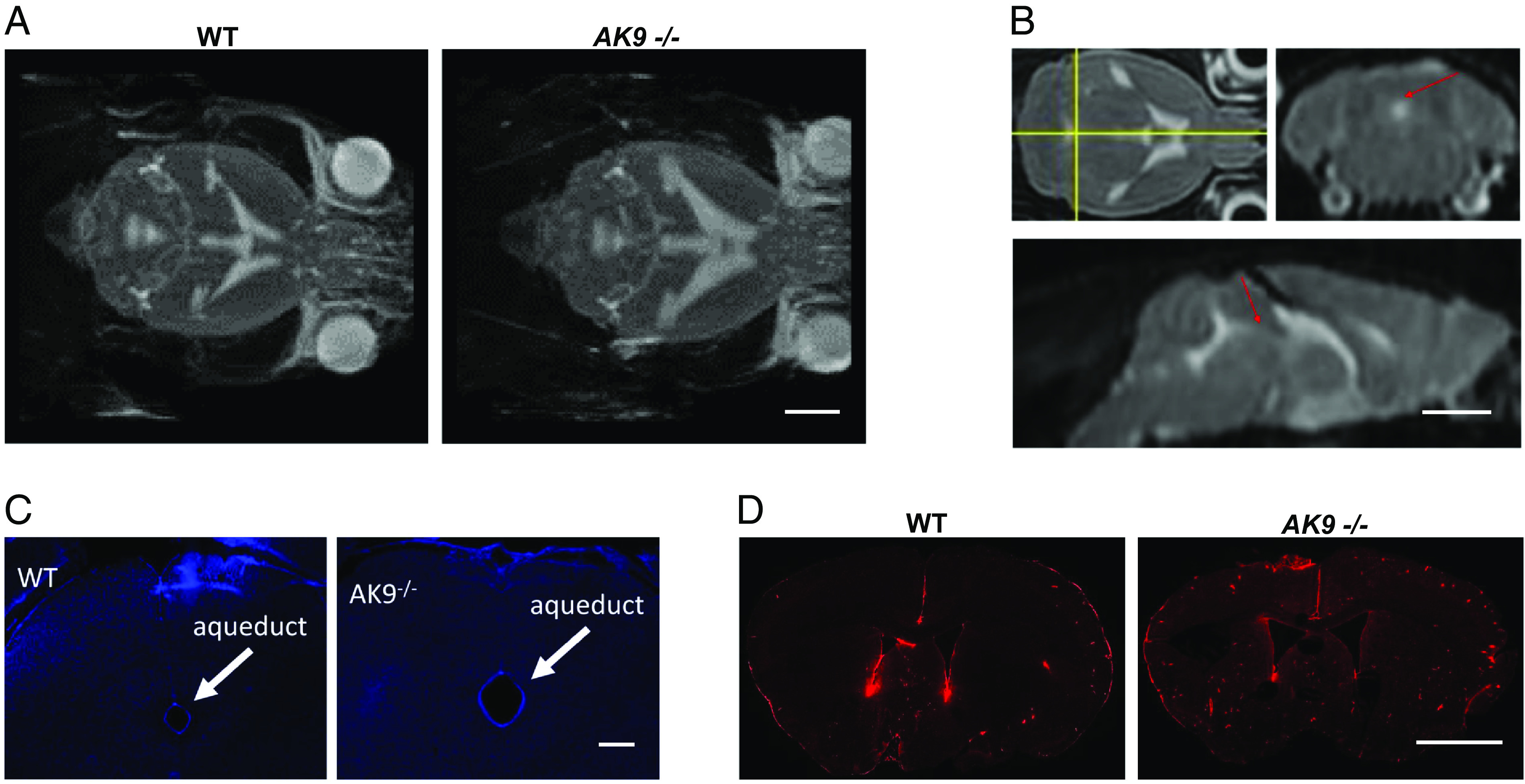
iNPH-associated *AK9* mutation causes a communicating hydrocephalus. (*A*) T2-weighted axial magnetic resonance (MR) images of mouse brains obtained from wild-type (WT) and *AK9*−/− mice showing the size of the lateral, third, and fourth ventricles. (Scale is 1 cm.) (*B*) T2-weighted axial, coronal, and sagittal MR images indicating the presence of CSF within the cerebral aqueduct and fourth ventricle (arrows). (Scale ~1 cm.) (*C*) Cryostat sections obtained from a wild-type (WT) and an *AK9*−/− mouse brain. The sections were stained with DAPI and viewed under epifluorescence to identify cell nuclei. Arrows point to the cerebral aqueduct, which is patent. Note the larger size of the aqueduct in the *AK9*−/− mouse. (Scale ~ 0.5 mm.) (*D*) Fluorescence micrographs of coronal sections of WT and AK9−/− mouse brains that were harvested 5 min after intraventricular injection of 70 kD fluorescent dextran (red). Note the presence of dextran in the subarachnoid and paravascular spaces. (Scale ~ 0.5 mm.)

We used MRI to assess ventricular size in *AK9* mutant mice. At birth, homozygous *AK9*−/− mice displayed larger ventricles than WT mice, while there was no difference in ventricular size between heterozygous *AK9*+/− mice and WT mice. By postnatal day seven (P7), male and female *AK9−/−* mice showed markedly enlarged ventricles when compared to wild-type mice, and this difference persisted beyond 9 mo of age (Fig. 6*A*). MRI examination of adult *AK9*−/− mouse brains (Fig. 6*B*) as well as visual inspection of the cerebral aqueduct in frozen sections of the mouse brain (Fig. 6*C*) revealed a patent cerebral aqueduct. Unilateral injection of 70 kD fluorescent dextran into the lateral ventricle confirmed that the cerebral aqueduct was functionally patent in both *AK9*+/− and *AK9*−/− mice (Fig. 6*D*). Notably, the 70 kD fluorescent dextran circulated throughout the ventricular system, subarachnoid spaces and paravascular pathways within 5 min in WT and *AK9*−/− mice, indicating rapid bulk flow of cerebrospinal fluid.

**Fig. 7.**
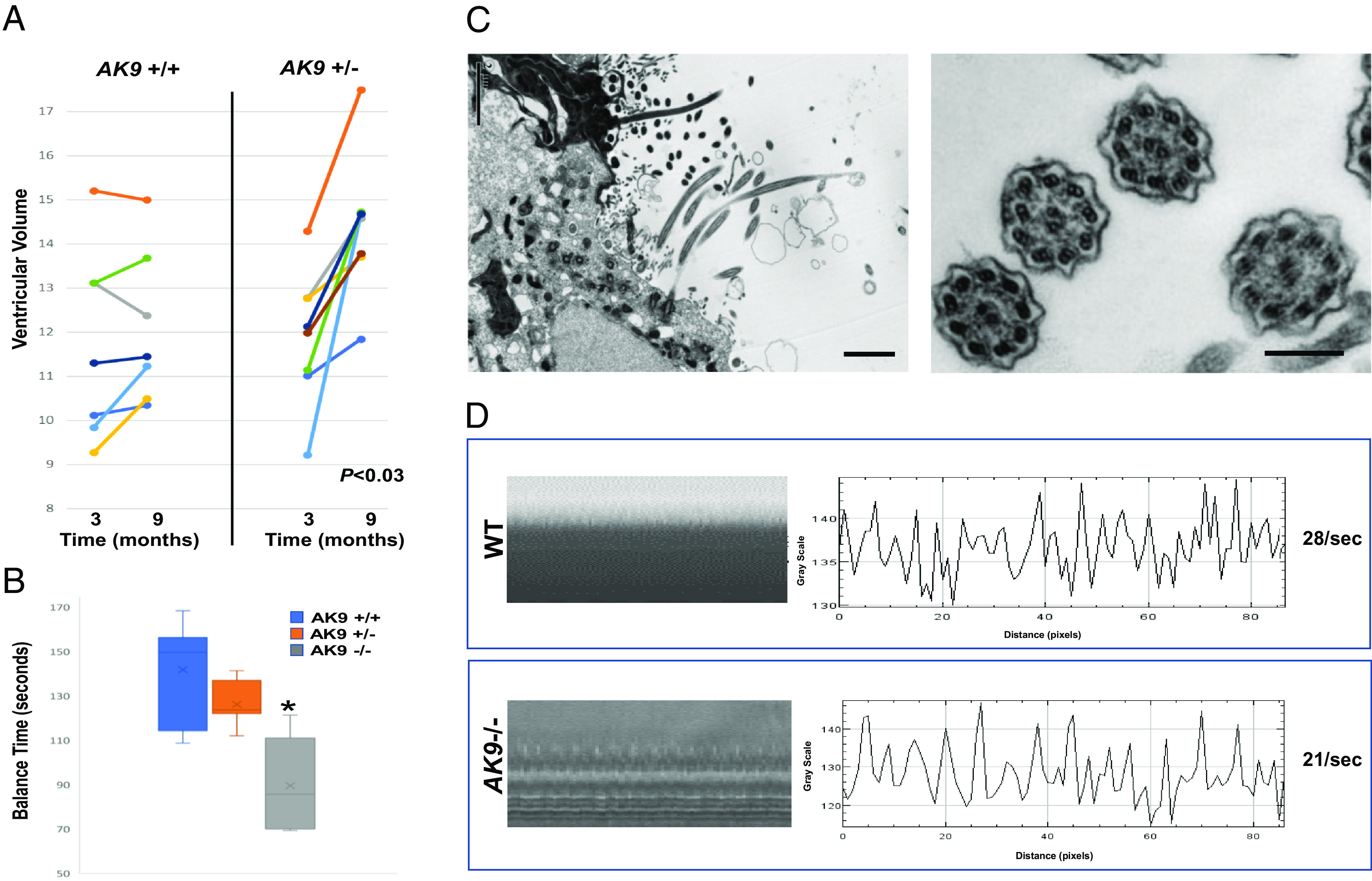
iNPH-associated *AK9* mutation causes age-dependent communicating hydrocephalus and decreases cilia beat frequency. (*A*) Ventricular volume in the brains of WT and *AK9*+/− mice was measured at 3 mo and again at 9 mo using T2-weighted MRI. (*B*) Rotarod data for WT (n = 7), *AK9*+/− (n = 7), and *AK9−/−* (n = 5) mice at 3 mo of age. *= *P* < 0.0023, unpaired *t* test. (*C*) Transmission electron micrographs of the mouse brain ependymal surface (*Left*) and ependymal motile cilia (*Right*) from *AK9*−/− mice. (*D*) Tracings showing high-speed video microscopy measurements of cilia beat frequency in acute ventricular explants obtained from WT and *AK9*−/− mice.

At 3 mo of age, ventricular volume in homozygous *AK9−/−* mice (n = 8) was about threefold greater than ventricular volume in WT mice (n = 6, *P* < 0.0001, unpaired *t* test). Ventricular volume in heterozygous *AK9*+*/−* mice (n = 8) was no different than that of WT mice at 3 mo, but was increased by approximately 20% when compared to the ventricular volume of WT mice at 9 mo (Fig. 7*A*, *P* < 0.03, unpaired *t* test). This was due to a greater age-dependent increase in ventricular volume between 3 mo and 9 mo in *AK9*+/− mice when compared to WT mice.

Gait and balance impairment is the most common symptom observed in iNPH patients. To assess balance in *AK9* mutant mice, we used the rotarod test. As mentioned previously, *AK9*−/− mice (four females and one male), displayed very large ventricles at 3 mo, while *AK9*+/− mice (seven males) had no signs of hydrocephalus at P7 or at 3 mo. Accordingly, *AK9*−/− mice displayed significantly decreased balance during rotarod testing at 3 mo when compared to WT mice at 3 mo (*P* < 0.0023, unpaired *t* test), while *AK9*+/− mice showed no statistical difference from WT mice during that same time period (*P* < 0.1116, unpaired *t* test, Fig. 7*B*). Although rotarod balance testing at 9 mo identified a subgroup of *AK9*+/− mice that showed a marked balance deficit when compared to WT mice, most *AK9*+/− mice displayed normal balance such that there was no statistical difference overall in terms of balance between WT (seven males) and *AK9*+/− mice (seven males).

Motile cilia are structurally and functionally similar to flagella, and mutations that disrupt flagellar function often have a similar effect on motile cilia. Importantly, loss of motile cilia function has been associated with hydrocephalus in animals and in humans. We therefore examined the effect of *AK9* mutation on ependymal motile cilia number, structure, and function. Transmission electron microscopy revealed a normal 9 + 2 axonemal structure with intact dynein arms in motile cilia from *AK9*−/− mice (Fig. 7*C*). To measure cilia length, cilia axonemes were physically isolated by allowing them to adhere to a poly-D-lysine-coated glass slide that was touched to the ventricular surface. On average, cilia from *AK9*−/− mice were 12% shorter than cilia from WT mice (WT, 12.5 ± 1.5 mm; AK9−/−, 11.2 ± 1.9 mm, *P* < 0.01, unpaired *t* test).

In light of the effect of *AK9* mutation on flagellar motility, we used high-speed video microscopy with interferometry to examine the effect of *AK9* mutation on motile cilia beat frequency (CBF) in acutely isolated mouse ventricular explants. When compared to wild-type mice, *AK9*+/− mice showed a 9% decrease in CBF, while *AK9*−/− mice showed a 25% decrease in CBF (WT, 28 ±   1.5/s; *AK9*+/*−*, 25.6 ±   2.4/s; *AK9−/−*, 20.9 ±   1.3/s. WT vs. *AK9*+/−, *P* < 0.05 and WT vs. *AK9−/−*, *P* < 0.01, unpaired *t* test) (Fig. 7*D*).

## Discussion

### Ventricular Multiciliated Epithelia as a Site of Origin for iNPH.

iNPH is a disorder of aging, occurring almost exclusively after the age of 60 in individuals who were previously asymptomatic. It is a common disorder, affecting 1% and 5% of individuals over the age of 60 and 75, respectively. Thus, a genetic underpinning of iNPH is unlikely to involve a single gene with an autosomal recessive pattern of inheritance because these are generally rare disorders. In fact, for the few reports of familial iNPH where the inheritance pattern has been identified, all were autosomal dominant. A recent report on a family with familial iNPH identified a heterozygous mutation in the cilia-associated protein, *CFAP43*, as a possible cause of chronic sinusitis and familial NPH in two affected family members, suggesting an autosomal dominant effect (14). Unfortunately, these investigators only studied the effects of *CFAP43* mutation in homozygous *CFAP43−*/*−* knockout mice; heterozygous *CFAP43*+/− mice were not examined to determine whether they also developed hydrocephalus. We recently reported that heterozygous mutations in *CWH43* are associated with iNPH in humans and in mice, consistent with an autosomal dominant mode of transmission (20). Here, we identify nine additional genes harboring multiple damaging heterozygous mutations that are statistically enriched among patients with iNPH. All of the iNPH-associated genes show high levels of expression in multiciliated choroid plexus and/or ependymal cells, and most of the encoded proteins have been linked to the function of cilia in other studies.

One of the iNPH-associated genes that has not been previously associated with cilia is a myosin heavy chain gene, *MYH13*. We identified heterozygous mutations in *MYH13* in a subset of iNPH patients. Although *MYH13* has been reported to be primarily expressed in extraocular and laryngeal muscles (32), in situ mRNA hybridization suggests that it is also expressed in brain ependymal and choroid plexus cells. Further studies are needed to determine the role of Myh13 in these cells.

We identified iNPH-associated heterozygous mutations in another myosin gene, *MYO7A*. *MYO7A* encodes the actin-associated motor protein, myosin VIIA (Myo7a), which has been detected in cilia from cochlear hair cells, olfactory neurons, renal distal tubules, respiratory epithelial cells and retinal pigmented epithelial cells, and photoreceptors (24). Myo7a participates in the apical localization of retinal pigment epithelium melanosomes and in the removal of apical RPE phagosomes. In photoreceptor cells, Myo7A functions as a selective barrier for membrane proteins at the distal end of the transition zone of the cilium (33). Mutations in *MYO7A* affect the development of retinal photoreceptors and the mechanosensory stereocilia of hair cells, thereby causing a progressive deafness/blindness syndrome called Usher Syndrome 1B. A literature search revealed a single report about three family members with Usher Syndrome 1B who also had a Dandy–Walker Malformation variant, which is characterized by fourth ventricular hydrocephalus (34).

We detected heterozygous mutations in *OTOG* in 9.6 percent of iNPH patients. *OTOG* encodes otogelin, a transmembrane protein related to secreted epithelial mucins that is localized to the fibrous horizontal top connectors that link the tectorial membrane to the adjacent outer hair cell stereocilia (35). Autosomal recessive mutations in *OTOG* cause congenital hearing impairment. We detected strong expression of *OTOG* mRNA and otogelin immunoreactivity in the choroid plexus and ependymal layer of the brain. Notably, mutations in both *OTOG* and *MYO7A* have been associated with deafness in humans and in mice, but only *MYO7A* mutations have been associated with hydrocephalus (24, 28, 33, 34). The role of otogelin in ventricular multiciliated epithelial cells remains to be determined.

*SPG11* encodes spatacsin, a transmembrane protein that regulates the autophagic lysosomal reformation process that is crucial for lysosomal homeostasis during autophagy. Complete loss of spatacsin function leads to defective autophagy, lysosomal lipid accumulation, and neurodegeneration (36). Homozygous mutations in *SPG11* cause hereditary spastic paraplegia (HSP), a genetic disorder characterized by progressive spasticity and paraparesis. Additional symptoms include cognitive impairment, parkinsonism, psychosis, peripheral neuropathy, and sphincter disturbance. A classic finding on MRI is thinning of the corpus callosum, but a majority of HSP patients also display enlarged ventricles (36). Here, we present evidence that heterozygous *SPG11* mutations are enriched among patients with shunt-responsive iNPH, that the *SPG11* gene shows high levels of expression in ventricular muticiliated epithelial cells, and that the protein encoded by *SPG11*, spatacsin, localizes to motile cilia. In the current study, none of the iNPH patients who were found to have heterozygous *SPG11* mutations presented with spasticity or peripheral neuropathy. Because all of the iNPH patients displayed ventriculomegaly and experienced symptomatic improvement after CSF drainage, our findings are in alignment with the observation that HSP patients display enlarged ventricles and raise the possibility that heterozygous *SPG11* mutations may contribute to iNPH.

*HAVCR1* encodes hepatitis A virus cellular receptor 1, which is also known as kidney injury molecule 1 (Kim1). Kim1 has been localized to cilia where it interacts with TRPP2 (a.k.a. polycystic kidney disease 2) and regulates intracellular calcium in response to flow in renal tubule epithelial cells (25). Expression of a mutant form of Kim1 that lacks a conserved tyrosine in the intracellular tail abolished the calcium increase in response to flow in a dominant negative manner.

Another iNPH-associated gene, *PRKD1*, encodes protein kinase D1 (PRKD1). Like Kim1, PRKD1 associates with TRPP2 (37). We identified strong PRKD1 immunoreactivity in ventricular epithelial cells. Neither PRKD1 nor Kim1 have been associated with hydrocephalus previously.

Several heterozygous mutations in *NOTCH1* were identified among iNPH patients. The Notch1 receptor is highly expressed in ependymal cells, and Notch signaling is required to maintain ependymal cells in a quiescent state (38). Inhibition of Notch signaling causes ependymal cells to re-enter the cell cycle and give rise to new neurons, although they have a limited capacity to do so. Ultimately, Notch inhibition leads to ependymal cell depletion (38). Several of the *NOTCH1* mutations identified here are located in the EGF-like and Notch domains. It will be important to determine whether these are indeed damaging mutations, as predicted in silico.

We observed recurrent mutations in *RXFP2* in about 7.5% of iNPH patients. Rxfp2 is a G-protein coupled receptor for insulin-like 3 (Insl3) and relaxin. The Rxfp2/Insl3/relaxin system has been implicated in the regulation of female reproduction, the cardiovascular system, bone growth, and other biological processes (39). The same mutation identified in the current study has been associated with cryptorchidism in mice, although whether it plays a role in human cryptorchidism remains controversial. Rxfp2 immunoreactivity is present in mammalian sperm, and the Rxfp2 ligand, relaxin, increases sperm motility (40). Here, we report evidence that the Rxfp2 receptor also localizes to ependymal motile cilia. Additional studies are underway to determine the effect of Rxfp2 activation on cilia structure and motility.

Many cases of congenital hydrocephalus are caused by biallelic mutations in genes affecting primary cilia. Because of their autosomal recessive nature, most genetic subtypes of inherited congenital hydrocephalus are quite rare, and many cases of genetically inherited congenital hydrocephalus are considered syndromic because they are accompanied by developmental abnormalities in the brain and/or other organs. This situation contrasts with that of iNPH, which is common among the elderly, and where most patients appear normal and are neurologically asymptomatic until the sixth decade of life. All of the iNPH-associated mutations identified in this study are heterozygous, consistent with the autosomal dominant mode of transmission that has been reported for several familial iNPH pedigrees. Our studies in mice harboring heterozygous iNPH-associated mutations in either *CWH43* or *AK9* show that these mutations can cause communicating hydrocephalus in an autosomal dominant manner. The autosomal dominant mode of transmission and multigenic nature of this disorder provide an explanation for why iNPH occurs at such a high frequency among the aging population (5).

### A Possible Role for Heterozygous AK9 Mutations in iNPH.

Sperm exhibit a greatly increased flagellar beat amplitude in the presence of ADP (41). ADP is thought to exert its effect on flagellar beating by increasing the binding affinity of dynein for the β-subtubule of the outer doublets. Ak9 is a mononucleoside and dinucleoside kinase that catalyzes the interconversion of ATP/ADP/AMP, GTP/GDP/GMP, and CTP/CDP/CMP. We find that Ak9 is highly expressed in sperm and in epithelial cells containing motile cilia, including those in the respiratory and reproductive tracts. *AK9*−/− mice are sterile. We observed a markedly decreased flagellar beat amplitude in sperm from *AK9*−/− mice, consistent with decreased ATP/ADP interconversion caused by diminished Ak9 activity.

Interestingly, two related adenylate kinases, Ak7 and Ak8, are also highly expressed in sperm and in multiciliated cells, and mutations in the genes that encode these adenylate kinases (*AK7* and *AK8*) cause hydrocephalus (42). However, the phenotype of mice lacking each of these related adenylate kinases differs in important ways. Homozygous *AK8* mutations lead to mild to moderate communicating hydrocephalus in mice by 3 wk of age, and loss of Ak8 function does not cause male infertility. In contrast, homozygous *AK7* mutations in mice cause ultrastructural defects in motile cilia, male infertility due to decreased sperm production, and severe congenital hydrocephalus leading to death before 8 wk of age (42). This pattern of severe congenital hydrocephalus leading to perinatal death is often the result of obstructive hydrocephalus, although that was not specifically determined in *AK7* mutant mice. We identified a single report of a homozygous *AK7* mutation in humans that caused a severe sperm motility defect (asthenozoospermia) and male infertility, but not rhinitis, respiratory symptoms, or hydrocephalus (43). Further investigation revealed a selective loss of Ak7 protein expression in sperm, but not in ciliated respiratory epithelia. Although ventricular ciliated cells were not examined in that study, the lack of hydrocephalus when compared to the severe hydrocephalus observed in *AK7* knockout mice suggests that this particular mutation may selectively reduce Ak7 protein expression in sperm but not in respiratory or ependymal cells.

Until now, the functional role of Ak9 in mammals was unknown. Mutations in *AK9* were previously identified in a mutagenic screen for genes that cause hydrocephalus in zebrafish (44). *AK9* mutations have been statistically associated with subfertility in bulls, but no functional evidence for a role for Ak9 in fertility had been reported (45). Another report suggested that intronic mutations in *AK9* may act as a disease modifier in combination with *RAPSN* mutations in limb-girdle congenital myasthenia, but causality or a mechanism of action was not determined (46). Here, we show that Ak9 regulates the motility of sperm and motile cilia in mammals and that *AK9* mutations can cause male infertility and communicating hydrocephalus.

We have now used genetically engineered mice to experimentally validate two of the most frequently mutated iNPH-associated genes identified using whole exome sequencing, i.e., *AK9* and *CWH43* (20). A damaging single nucleotide variant was found in one of these two genes in nearly 25% of our iNPH patient cohort. Mice that are heterozygous for iNPH-associated *CWH43* or *AK9* mutations develop an iNPH-like syndrome that is characterized by grossly normal appearance and behavior, normal lifespan, age-dependent communicating hydrocephalus, and impairments of gait or balance. In the current study, we focused on the effect of the iNPH-associated *AK9* mutation on gait and balance because gait difficulty is the most frequently observed symptom in patients with iNPH. Additional experiments examining the effects of this mutation on cognitive impairment and urinary incontinence will be helpful in determining whether this iNPH mouse model replicates these other commonly observed iNPH symptoms. As mentioned previously, iNPH-associated mutations identified in this study affected two other genes, *MYO7A* and *SPG11*, that have previously been shown to cause hydrocephalus or ventriculomegaly. Interestingly, we and others have reported that symptoms such as chronic sinusitis (14) or stuttering (47) can occur early in life and precede the classical late-onset symptoms of gait instability, incontinence, and cognitive impairment observed in iNPH patients. These observations suggest that previously unrecognized nonclassical symptoms of iNPH may develop before the sixth decade of life, and they are consistent with studies suggesting that asymptomatic ventricular enlargement may precede symptom onset in iNPH patients (48).

All of the iNPH-associated genes identified in the current study (including *AK9*) show high levels of expression in ependymal cells, and most of the encoded proteins have been linked to cilia function in other studies. We find that iNPH-associated mutations affecting two of these genes, *AK9* and *CWH43*, alter ependymal motile cilia function and cause an iNPH-like syndrome in mice that is characterized by communicating hydrocephalus and balance impairment. Taken together, our findings support the notion that genetic traits that alter the function of ependymal cells may predispose to the development of iNPH. However, *AK9, CWH43,* and several other iNPH-associated genes are expressed in other cell types in the brain and elsewhere. To conclusively demonstrate that defects in ependymal cell function cause iNPH, it would be helpful to introduce iNPH-associated gene mutations specifically in ependymal cells using tissue-specific gene editing or conditional knockin mice.

## Supplementary Material

Appendix 01 (PDF)

## Data Availability

The sequencing data for this study have been deposited in dbGaP (22).
