# Supplementary material for: A role for mutations in AK9 and other genes affecting ependymal cells in idiopathic normal pressure hydrocephalus
Source: Proc Natl Acad Sci U S A. 2023 Dec 15;120(51):e2300681120. doi: 10.1073/pnas.2300681120 (PMC10743366; doi:10.1073/pnas.2300681120)

Mutations in *AK9* and Other Genes Affecting Ependymal Cells Cause Idiopathic Normal Pressure Hydrocephalus

Hong Wei Yang[1,3,4], Semin Lee[3,4], Bethany Berry[1], Dejun Yang[1], Shaokuan Zheng[1], Rona S. Carroll[1,3,4], Peter J. Park[3,4] and Mark D. Johnson[1,2,3,4*]

Supplementary Data

Supplementary Fig. 1: Genotype/phenotype correlations among genetic subsets of iNPH patients. Upper panel illustrates pattern of mutations occurring in three independent sets of 20 patients (gray boxes, top row), 12 patients (green boxes, top row) and 21 patients (yellow boxes, top row). Each vertical column displays the mutation status for the 10 NPH-associated genes in an individual patient. Pink boxes identify mutated genes. Only genes with at least 3 deleterious, statistically significant mutations are shown. Lower panel correlates these mutations with a series of clinical and demographic factors in this patient population. Lower panel color scheme: White = Not affected; Light blue = Mild; Medium blue = Moderate; Dark blue = Major or Severe.

Supplementary Fig. 2: Schematic diagram illustrating the domain structure and location of mutations in proteins encoded by iNPH-associated genes.

Supplementary Fig. 3: Expression of *AK9* mRNA isoforms in mouse tissues. RT-PCR was used to analyze *AK9* mRNA isoform expression in mouse whole brain, ependymal layer, muscle, liver, kidney, trachea and testis.

Supplementary Fig. 1

GENE	15-14	109-14	2-13A	92-14	86-15	142-14	161-14	133-14	132-14	103-14	11-15	01-16	119-14	104-14	93-14	65-14	02-16	18-13	83-15	148-14	28-13	110-14	131-14	53-15	04-138	169-14	99-15	77-14	98-15	70-15	90-15	66-14	79-15	96-15	09--13	48--14	89-15	141-14	17--13	26-15	55--14	23-14	61-15	122-14	47--14	117-14	49-15	05-16	122-14	34-15	77-15	87-15	95-15
CWH43	1	1	1	1	0	0	0	0	0	0	0	0	0	0	0	0	0	0	0	0	0	0	0	0	0	0	0	0	0	0	0	0	0	0	0	0	0	0	0	0	0	0	0	0	0	0	0	0	0	0	0	0	0
RXFP2	0	0	0	1	1	1	1	0	0	0	0	0	0	0	0	0	0	0	0	0	0	0	0	0	0	0	0	0	0	0	0	0	0	0	0	0	0	0	0	0	0	0	0	0	0	0	0	0	0	0	0	0	0
OTOG	0	0	0	0	0	0	1	1	1	1	1	0	0	0	0	0	0	0	0	0	0	0	0	0	0	0	0	0	0	0	0	0	0	0	0	0	0	0	0	0	0	0	0	0	0	0	0	0	0	0	0	0	0
PRKD1	0	0	0	0	0	0	0	0	0	0	1	1	1	1	0	0	0	0	0	0	0	0	0	0	0	0	0	0	0	0	0	0	0	0	0	0	0	0	0	0	0	0	0	0	0	0	0	0	0	0	0	0	0
MYH13	0	0	0	0	0	0	0	0	0	0	0	0	0	1	1	1	1	0	0	0	0	0	0	0	0	0	0	0	0	0	0	0	0	0	0	0	0	0	0	0	0	0	0	0	0	0	0	0	0	0	0	0	0
HAVCR1	0	0	0	0	0	0	0	0	0	0	0	0	0	0	0	0	0	1	1	1	1	0	0	0	0	0	0	0	0	0	0	0	0	0	0	0	0	0	0	0	0	0	0	0	0	0	0	0	0	0	0	0	0
AK9	0	0	0	0	0	0	0	0	0	1	1	0	0	0	0	0	0	0	0	0	0	1	1	1	0	0	0	0	0	0	0	0	0	0	0	0	0	0	0	0	0	0	0	0	0	0	0	0	0	0	0	0	0
SPG11	0	0	0	0	0	0	0	0	0	0	0	0	0	0	0	0	0	0	0	0	0	0	0	1	1	1	1	0	0	0	0	0	0	0	0	0	0	0	0	0	0	0	0	0	0	0	0	0	0	0	0	0	0
MYO7A	0	0	0	0	0	1	1	0	0	0	0	0	0	0	0	0	0	0	0	0	0	0	0	0	0	0	1	1	0	0	0	0	0	0	0	0	0	0	0	0	0	0	0	0	0	0	0	0	0	0	0	0	0
NOTCH1	0	0	0	0	0	0	0	0	0	0	0	0	0	0	0	0	0	0	0	0	0	0	0	0	0	0	0	0	1	1	1	1	0	0	0	0	0	0	0	0	0	0	0	0	0	0	0	0	0	0	0	0	0
AGE (yrs)	76	84	74	65	70	75	76	84	81	77	72	68	81	80	68	76	75	76	75	73	70	69	75	77	80	76	72	89	73	70	78	68	77	75	77	81	76	81	79	68	67	76	81	75	84	86	74	79	70	70	72	78	64
Gender	F	F	M	F	F	M	F	F	F	M	F	M	F	F	M	F	F	F	F	F	F	M	F	M	F	M	F	M	M	F	M	M	M	M	F	M	M	F	M	F	M	M	F	F	F	M	M	F	M	F	F	M	M
Sx Duration (yrs)		3	4	4	2	2	2	2	1	6	1	2	1	2		2	2	2	1	4	2	2	2	20	5	2	1	10		2	2	3	3	2	2	1		1		3	1				2	3	2	1	2	1	1	1	1
Gait Difficulty																																																					
Urinary Sxs																																																					
Cognitive Difficulty																																																					
Hearing Difficulty																																																					
Vision Loss																																																					
Speech Difficulty																																																					
Neuropathy																																																					
Spasticity																																																					
Tremor																																																					
Hypertension																																																					
Diabetes																																																					
Hyperlipidemia																																																					
Alcohol Use																																																					
Degree Improved																																																					

Supplementary Fig. 2

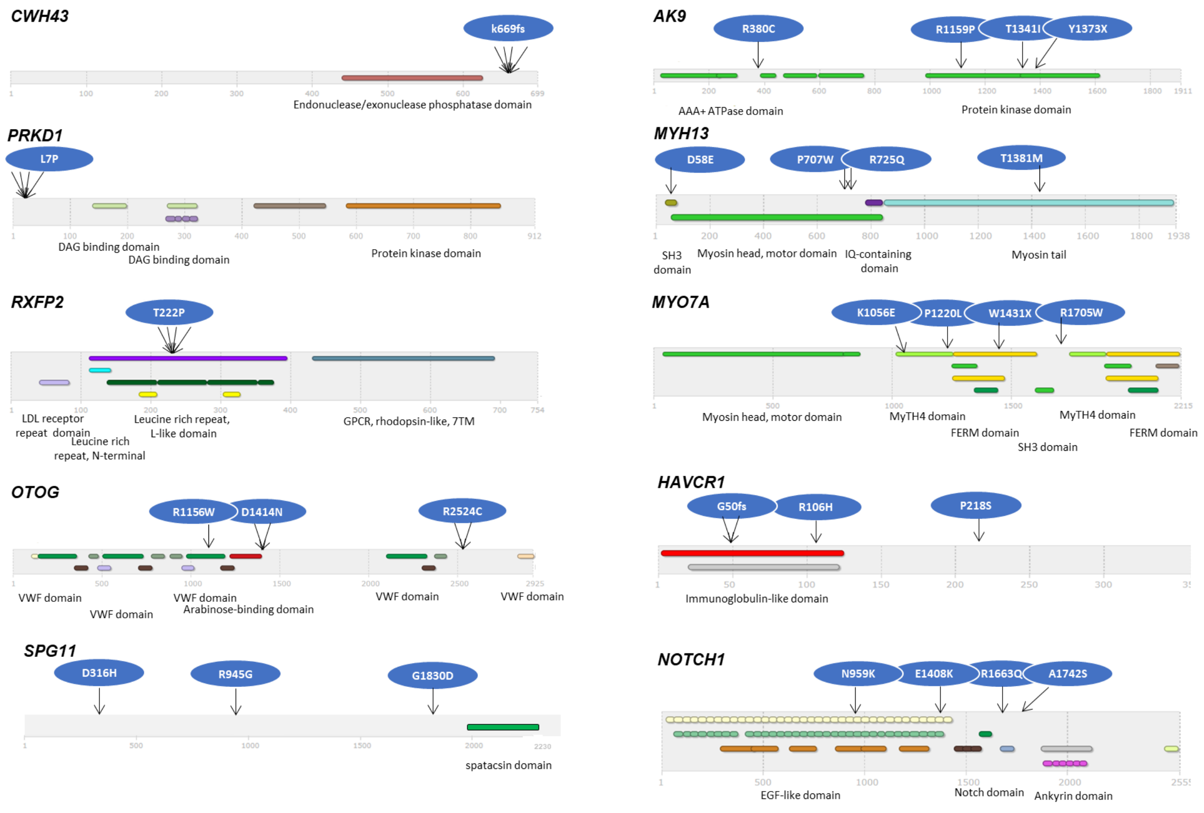

Supplementary Fig. 3

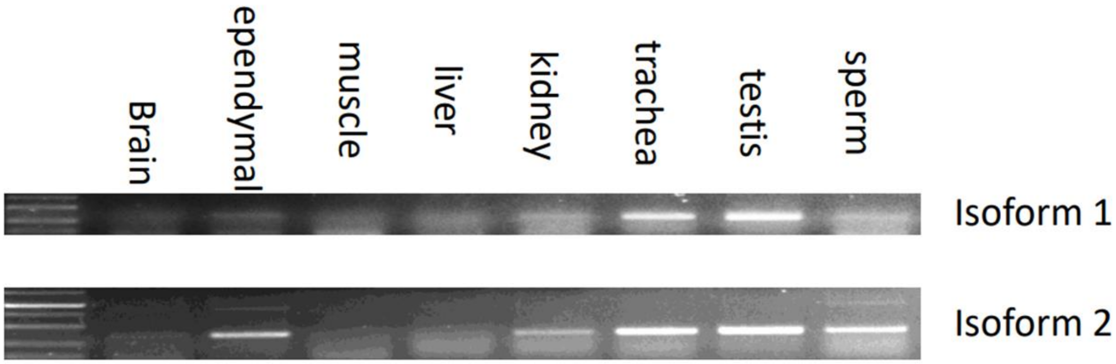

Supplement: Supplementary file 1 — Appendix 01 (PDF) [file pnas.2300681120.sapp.pdf]
